# Comparative Study of the Fatty Acid Binding Process of a New FABP from *Cherax quadricarinatus* by Fluorescence Intensity, Lifetime and Anisotropy

**DOI:** 10.1371/journal.pone.0051079

**Published:** 2012-12-21

**Authors:** Jiayao Li, Etienne Henry, Lanmei Wang, Olivier Delelis, Huan Wang, Françoise Simon, Patrick Tauc, Jean-Claude Brochon, Yunlong Zhao, Eric Deprez

**Affiliations:** 1 Laboratoire de Biologie et Pharmacologie Appliquée (LBPA), CNRS UMR8113, Ecole Normale Supérieure Cachan, Institut d'Alembert, Cachan, France; 2 School of Life Science, East China Normal University, Shanghai, China; University of South Florida College of Medicine, United States of America

## Abstract

Fatty acid-binding proteins (FABPs) are small cytosolic proteins, largely distributed in invertebrates and vertebrates, which accomplish uptake and intracellular transport of hydrophobic ligands such as fatty acids. Although long chain fatty acids play multiple crucial roles in cellular functions (structural, energy metabolism, regulation of gene expression), the precise functions of FABPs, especially those of invertebrate species, remain elusive. Here, we have identified and characterized a novel FABP family member, Cq-FABP, from the hepatopancreas of red claw crayfish *Cherax quadricarinatus*. We report the characterization of fatty acid-binding affinity of Cq-FABP by four different competitive fluorescence-based assays. In the two first approaches, the fluorescent probe 8-Anilino-1-naphthalenesulfonate (ANS), a binder of internal cavities of protein, was used either by directly monitoring its fluorescence emission or by monitoring the fluorescence resonance energy transfer occurring between the single tryptophan residue of Cq-FABP and ANS. The third and the fourth approaches were based on the measurement of the fluorescence emission intensity of the naturally fluorescent cis-parinaric acid probe or the steady-state fluorescence anisotropy measurements of a fluorescently labeled fatty acid (BODIPY-C16), respectively. The four methodologies displayed consistent equilibrium constants for a given fatty acid but were not equivalent in terms of analysis. Indeed, the two first methods were complicated by the existence of non specific binding modes of ANS while BODIPY-C16 and cis-parinaric acid specifically targeted the fatty acid binding site. We found a relationship between the affinity and the length of the carbon chain, with the highest affinity obtained for the shortest fatty acid, suggesting that steric effects primarily influence the interaction of fatty acids in the binding cavity of Cq-FABP. Moreover, our results show that the binding affinities of several fatty acids closely parallel their prevalences in the hepatopancreas of *C. quadricarinatus* as measured under specific diet conditions.

## Introduction

Fatty acid-binding proteins (FABPs) are small cytosolic proteins (126–134 amino acids; 14–15 kDa) which are present in both vertebrates and invertebrates and have the ability to bind to long chain fatty acids [1]–[3]. All FABPs belong to the lipid-binding protein (LBP) superfamily, which also includes the cellular retinoic acid- and retinol-binding proteins (CRABP and CRBP, respectively) as well as P2 myelin proteins, adipocyte LBP and mammary-derived growth inhibitors [4]. They share the same overall tertiary structure, comprising 10 anti-parallel β-strands forming a β-clam, which together with two α-helices, delimitate an inner cavity corresponding to the binding site of hydrophobic ligands [4], [5]. Entry of fatty acids into the binding cavity occurs via the portal region located on the surface of the protein [6].

Since the first mammalian FABP was reported in 1972 [7], many types of FABPs have been identified in vertebrate species and their names depend on the tissue corresponding to the first isolation/identification (liver, heart, intestine, adipocyte, myelin, brain…) [3], [8]. Most of them are well characterized at the structural level (X-ray and NMR structures) and the binding specificity of FABPs is relatively well established [4], [9], [10]. In vertebrate species, FABPs isolated from the same tissue consistently display high sequence identities (>70%) whereas FABPs from different tissues share low sequence identities in a given species, ranging from 20% to 70% [10]. FABPs are involved in the uptake and transport of fatty acids from the plasma membrane to intracellular sites of conversion, a process which is fundamental for the modulation of cell growth and proliferation [3], [9]. Some of them also display antioxidant activities [11]. However, due to the plurality of roles of long chain fatty acids (structural, energy metabolism, regulation of gene expression [12]–[14]), the diversity of FABPs, their wide tissue distributions and their ability to bind a wide range of ligands, the precise biological function and the physiological role of each type of FABP remain imperfectly understood. The situation is even more complicated considering that specialized functions for each FABP probably depend on their tissue-specific locations.

Regarding the invertebrate species, the number of identified FABPs is only about 50 since the first invertebrate FABP was identified in the desert locust *Schistocerca gregaria* [15]. Whereas a large number of vertebrate FABPs have been extensively studied phylogenetically and for ligand-binding specificities, relatively less information is available regarding invertebrate FABPs. Recently, molecular biology, gene expression profile and structural studies have substantially increased the information related to the evolutionary and the cellular diversity of invertebrate FABPs [1], [2], [16], [17]. Invertebrate FABPs display low sequence identities with vertebrate FABPs, even though a rather modest but significantly higher sequence identity exists with the H-FABP type (Heart) [1], [18], [19]. Taking into account that the vertebrate H-FABP group has a wide distribution and multiple functions (in contrast to other groups which are more specialized), one can reasonably assume that invertebrate FABPs may also ensure a wide spectrum of biological functions [1], [20]. However, our knowledge about the physiological role(s) of invertebrates FABPs remains strongly limited and only little information is available regarding fatty acid binding properties of the invertebrate LBP subgroup. Until now, such studies are restricted to a few species such as *Schistocerca gregaria*, *Locusta migratoria*, *Metapenaeus ensis*, *Manduca sexta, Eriocheir sinensis* [15], [17], [21], [22].

As a major energy source and a structural element of phospholipid membranes, fatty acids play an important role during the development of decapod crustaceans. At the reproduction stage, the red claw crayfish *Cherax quadricarinatus* requires a large amount of fatty acids to meet the need of gonad development, some essential fatty acids have also been shown to be crucial for gonad maturation and brood quality [23]. In the present work, a new FABP gene was cloned from the hepatopancreas of *C. quadricarinatus* and the corresponding recombinant protein, Cq-FABP, was expressed in *Escherichia coli* and purified. We report here the characterization of ligand-binding affinity of Cq-FABP by four different fluorescence-based approaches. In all approaches, fatty acid binding properties were investigated by competitive binding assays. In the two first approaches, the classical fluorescent probe 8-Anilino-1-naphthalenesulfonic (ANS), which binds to internal binding cavities of protein, was used either by directly monitoring its fluorescence emission or by monitoring the fluorescence resonance energy transfer (FRET) occurring between the single tryptophan residue of Cq-FABP and ANS. We show that, using these two approaches, data analysis must be conducted with caution particularly due to a strong propensity of ANS to bind to secondary binding sites of Cq-FABP. By contrast, the third and the fourth approaches, using either the steady-state fluorescence intensity of the naturally fluorescent cis-parinaric acid probe or the steady-state fluorescence anisotropy of a fluorescently labeled fatty acid (BODIPY-C16), respectively, did not suffer from above-mentioned analytical difficulties - *i.e.* due to the existence of “non specific” binding modes - and are then appropriate for a systematic comparison of fatty acid binding properties. Our results show that the binding affinities of several fatty acids (palmitic acid (PA), oleic acid (OA), linoleic acid (LA), cis-5,8,11,14,17-eicosapentaenoic acid (EPA) and cis-4,7,10,13,16,19-docosahexaenoic acid (DHA)) strongly parallel their prevalences as previously measured under specific diet conditions in the hepatopancreas of *C. quadricarinatus* [23].

## Materials and Methods

### Cloning of the full-fength Cq-FABP cDNA

The cDNA encoding the entire *Cherax quadricarinatus* fatty acid binding protein (Cq-FABP) was cloned from its hepatopancreas. Total RNA was isolated from the hepatopancreas of red claws (intermolt period) using Trizol reagent according to the manufacturer's instructions (Invitrogen, Carlsbad, CA, USA). Total RNA (700 ng) was first converted into cDNA by reverse transcription using a Super-Script First-Strand Synthesis System for RT-PCR Kit (Invitrogen, Carlsbad, CA, USA). To amplify a fragment of Cq-FABP, a pair of degenerate PCR primers (Cq-FABP-PF1: 5′-GCA YTN GGC GTC GGN ATG ATG-3′ and Cq-FABP-PR1: 5′-YTG NGT GTC NGT GAA YTC YCT-3′) were designed based on the conserved amino acids sequences of FABPs from other crustacean species. PCR amplification was carried out as follows: 94°C for 5 min, 30 cycles of [94°C for 30 s, 55°C for 30 s, 72°C for 30 s] and 72°C for 10 min in an Applied Biosystems 2720 thermal cycler. A PCR product of 280 bp was gel-purified and ligated into the pGEM-T easy vector (Promega, Madison, WI, USA) and positive clones containing inserts of the predicted size were sequenced.

5′-RACE (5′-CTT GCT GGT CTG ACC CAA CTT CCT-3′) and 3′-RACE (5′-GAC AGC AGT CAA GGG CAA GAG TG-3′) primers were then designed for sequencing the flanking regions of the 280-bp fragment. The total RNA was isolated as mentioned above and mRNA was purified using the Oligotex mRNA kit (QIAGEN, Valencia, CA, USA) according to the protocol supplied by the manufacturer. The 5′- and 3′-RACE-Ready cDNA were produced by using the SMART RACE cDNA Amplification Kit (CLONTECH) according to the manufacturer's instructions with some modifications. Briefly, for the 5′-RACE-Ready cDNA synthesis, a 5 µl mixture containing 1 µg mRNA, 5′-CDS primer, SMART II A oligonucleotide was incubated for 2 min at 70°C and put on ice for 2 min. 2 µl of 5× First-Strand buffer, 1 µl of 20 mM DTT, 1 µl of 10 mM dNTP Mix and 1 µl of MMLV Reverse Transcriptase were then added (final volume, 10 µl). After incubation for 90 min at 42°C, 250 µl of Tricine-EDTA buffer was added and the mixture was further incubated for 7 min at 72°C. The synthesis of the 3′-RACE-Ready cDNA was conducted by using the same procedure as described above for the 5′-RACE-Ready cDNA, except that another special primer was used (3′-CDS primer) in the absence of the SMART II A oligonucleotide. Finally, both 3′- and 5′-RACE-Ready cDNA were amplified in a 50-µl mixture containing 2.5 µl of 3′- or 5′-RACE-Ready cDNA, 1 µl of 3′-RACE or 5′-RACE (10 µM), 5 µl of UPM (10×), 5 µl of Advantage 2 PCR buffer (10×), 1 µl of dNTP Mix (10 mM), 1 µl of Advantage 2 Polymerase Mix (50×) and 34.5 µl of PCR-Grade Water. PCR amplification was carried out as follows: 94°C for 4 min, 25 cycles of [94°C for 30 s, 60°C for 30 s, 72°C for 1 min] and 72°C for 10 min. PCR products were gel-purified and sequenced, allowing the determination of the Cq-FABP ORF.

A pair of specific primers was then designed to amplify the Cq-FABP cDNA by PCR: a Nde I-tailed upstream primer (5′-CAT ATG GCT CCC ATC GCA GGC AAA T-3′) and a BamH I-tailed downstream primer (5′-GGG ATC CTT ACT GGC GCT TGT AGA TGC GC-3′). The amplified Cq-FABP DNA was cloned in the pGEM vector (Promega, Madison, WI, USA) to generate the pGEM-Cq-FABP vector, allowing the determination of the Cq-FABP full-length cDNA sequence. This sequence and the deduced amino acid sequence were compared with other sequences reported in NCBI's GenBank using the BLAST program.

### Real-time quantitative RT-PCR (qRT-PCR)

**Figure 1:**
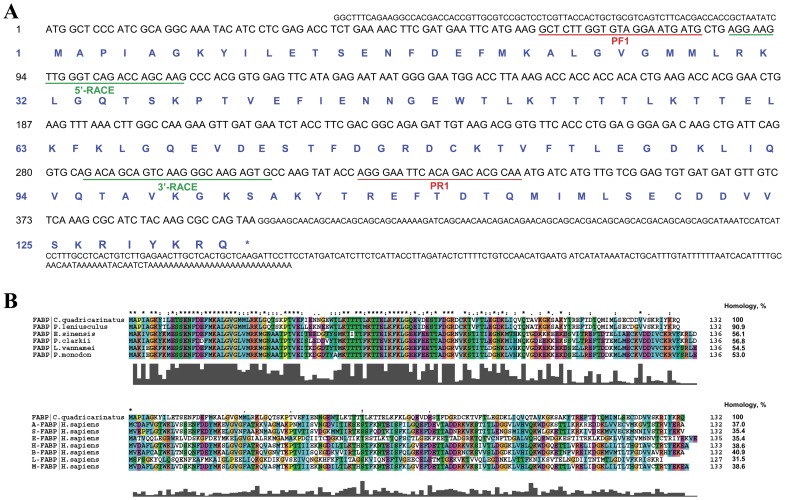
Nucleotide and amino acid sequences of Cq-FABP. The cDNA encoding the entire *Cherax quadricarinatus* fatty acid binding protein (Cq-FABP) was cloned from its hepatopancreas as described in Materials and Methods. DNA sequence and the deduced amino acid sequence are shown in **panel A**. Underlined sequences correspond to hybridization regions of degenerate PCR primers (red; PF1 and PR1, respectively) and primers used for the RACE PCR (green; 5′-RACE and 3′-RACE, respectively). (**B**) Sequence alignment of Cq-FABP with other invertebrate (top) and human FABPs (bottom). The percentages of homology are indicated on the right of the sequence alignments. Multiple sequence alignment was performed using the Clustal ×2.0 program. Human FABPs: A, adipocyte; S, serum; E, epidermal; H, heart; B, brain; L, liver; M, muscle.

Total RNA was isolated from hepatopancreas, ovary, intestine, antennal gland, hemolymph, gills, stomach and testis as above-described. Total RNA (700 ng) was reverse transcribed using a Super-Script First-Strand Synthesis System and RT-PCR kit (Invitrogen, CA, USA). Gene-specific primers for Cq-FABP (5′-TCA AGA CAG TGA GGC AAA G-3′ and 5′-GCA TCT ACA AGC GCC AGT AA-3′) were designed based on the sequence shown in Fig. 1A. Primers for β-actin mRNA were 5′-GTC CCC GTG TAT GAA GGT TT-3′ and 5′-GCT GTG GTG GTG AAG GAG TAG-3′. qRT-PCR was performed using TaKaRa SYBR Premix EX Taq reagent (TaKaRa, Dalian, China) and the Mx3000p Real Time PCR System (Stratagene, CA, USA) as previously described [23]. The amount of FABP mRNA was normalized using β-actin mRNA.

### Bacterial expression and purification of Cq-FABP

The restriction fragment obtained from Nde I and BamH I digestion of pGEM-Cq-FABP was then ligated into the expression vector pET15b (Novagen, Madison, WI, USA) with a 6 His-tagged sequence at the N-terminal end of the coding sequence. The resulting plasmid pET15b-His_6_-Cq-FABP was transformed into *Escherichia coli* BL21(DE3) strain (Stratagene, La Jolla, CA, USA). The bacteria were grown overnight in 20 ml LB medium containing 100 µg/ml ampicillin, and then diluted (1/500) into 1 liter of LB medium supplemented with 100 µg/ml ampicillin (the bacteria were grown in a 5-liter shaker flask). At an OD_600 nm_ of 0.6–0.8, protein expression was induced in bacterial cultures by the addition of 1 mM IPTG. *E. coli* cultures were then further incubated for 4 hours. After centrifugation (1,600 g for 30 min at 4°C), the cell pellet was resuspended in ice-cold buffer A [50 mM Tris pH 7.5, 10 mM imidazole, 100 mM NaCl and 4 mM β-mercaptoethanol, supplemented with a Complete™ Protease Inhibitor Cocktail (Roche, Mannheim, Germany)], and lysed using a French Press. After centrifugation (10,000 g for 30 min at 4°C), the supernatant was filtered using a 0.45 µm filter and incubated overnight at 4°C with Ni-NTA agarose beads (Qiagen, Courtaboeuf, France). The beads were washed twice with 20 mL of buffer A, and then, with buffer A supplemented with increasing concentrations of imidazole, from 40 to 700 mM (10 mL). The His-tagged Cq-FABP was eluted with buffer A supplemented with 1 M imidazole and dialyzed overnight against buffer B (20 mM Tris pH 7.5, 100 mM NaCl, 4 mM β-mercaptoethanol and 10% (v/v) glycerol). The protein was analyzed by a 15% SDS-PAGE gel and was pure (>95%) as judged by Coomassie staining. Protein concentration was measured by the Bradford method. The protein samples were aliquoted and rapidly frozen at −80°C. Cq-FABP preparations were systematically delipidated using Lipidex-1000 (Perkin Elmer, France), prior to binding assays. Lipidex-1000 removed protein-bound or free fatty acids at 37°C according to [24]. Delipidation was performed according to previous studies with modifications [25], [26]. Briefly, Cq-FABP was incubated at 37°C with 50% (w/v) Lipidex-1000 in 20 mM Tris pH 7.5, 100 mM NaCl. After centrifugation (4 min at 10,000 g), the supernatant was collected and the protein concentration was measured by the Bradford assay. Regarding incubation time with Lipidex-1000, three protocols were applied: (i) 1×15 min; (ii) 1×1 hour; (iii) 3× 10 min (each time using fresh Lipidex-1000). No significant difference in the binding properties of Cq-FABP was evidenced between the three protocols (tested using BODIPY-C16 in the anisotropy-based assay (see below)).

### ANS, DAUDA and fatty acids

8-Anilino-1-naphthalenesulfonic (ANS) acid ammonium salt and 11-((5-dimethylaminonaphthalene-1-sulphonyl)amino)-undecanoic acid (DAUDA) were obtained from Sigma Aldrich (St. Louis, MO, USA) (Molar extinction coefficients in H_2_O: ε_350 nm_ = 5,000 M^−1^.cm^−1^ and ε_335 nm_ = 5,000 M^−1^.cm^−1^, respectively). Palmitic acid (PA), oleic acid (OA), linoleic acid (LA), cis-5,8,11,14,17-eicosapentaenoic acid (EPA) and cis-4,7,10,13,16,19-docosahexaenoic acid (DHA) were obtained from Sigma Aldrich (St. Louis, MO, USA). Cis-parinaric acid was from Life Technologies (France) (Molar extinction coefficients in H_2_O: ε_320 nm_ = 70,000 M^−1^.cm^−1^). 4,4-difluoro-5, 7-dimethyl-4-bora-3a, 4a-diaza-s-indacene-3-hexadecanoic acid (BODIPY-C16 or C16) was from Invitrogen (Cergy Pontoise, France). All fatty acids were stored at −20°C. ANS was dissolved in buffer C (20 mM Tris pH 7.5, 100 mM NaCl). Cis-parinaric acid and non-fluorescent fatty acids were dissolved in ethanol; the final concentration of ethanol did not exceed 1% (v/v) in binding assays with cis-parinaric acid (0.7% (v/v) in binding assays with other fatty acids). DAUDA and BODIPY-C16 were dissolved in DMSO (final concentrations of DMSO: <0.1% (v/v) and = 10% (v/v) in binding assays with DAUDA and C16, respectively). All experiments described below were conducted in buffer C unless otherwise specified.

### Titration experiments of non-fluorescent fatty acids using ANS: Steady-state fluorescence intensity measurement setup

Fluorescence emission spectra were measured at 25°C on a Cary-Eclipse spectrofluorimeter (Varian, CA, USA) equipped with a thermostated cell holder, using solutions (70 µl) placed in microcuvettes (pathlength, 0.3 cm). The binding of non-fluorescent fatty acids (PA, OA, LA, EPA and DHA) to Cq-FABP was measured indirectly by equilibrium displacement experiments using ANS as a fluorescent probe, either by measuring the fluorescence emission of ANS or by measuring the FRET between tryptophan and ANS, resulting in the quenching of donor fluorescence. The excitation wavelength was then set at 296 or 350 nm for tryptophan or ANS, respectively; the slit widths and PMT voltage were adjusted to optimize the fluorescence signal at 25°C. For titration experiments, the fluorescence emission spectrum of tryptophan or ANS was measured and the integrated fluorescence signal of tryptophan (315–360 nm) or ANS (440–600 nm) was calculated. For samples containing high concentrations of ANS, the inner-filter effect was not negligible and was corrected [27]. UV-visible absorption spectra were carried out on a Uvikon XL spectrophotometer.

The equilibrium dissociation constant (K_d,FA_) relative to the binding of fatty acids (FA) to Cq-FABP was determined by competition experiments using ANS displacement and linear regression of the following relationship:
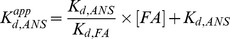
(1)where K_d,ANS_ is the dissociation constant relative to the binding of ANS to the fatty acid binding site, K_d_^app^_,ANS_ is the apparent dissociation constant of ANS obtained in the presence of a given fatty acid concentration.

### Titration experiments of non-fluorescent fatty acids using either cis-parinaric acid or DAUDA

Cis-parinaric acid and DAUDA were characterized by fluorescence emission in the 380–500 nm region (λ_max,em_: 410 nm) and in the 480–650 nm region (λ_max_,_em_: 540 nm), respectively (excitation wavelengths were set at 320 or 335 nm for cis-parinaric acid or DAUDA, respectively). The fluorescence intensity enhancement upon binding to Cq-FABP was monitored on a Cary-Eclipse spectrofluorimeter as indicated above. All the fluorescence emission intensities were corrected for the inner-filter effect. The emission intensity of a given fluorophore in the presence of Cq-FABP was also systematically corrected for the emission intensity of the free fluorophore. The calculation of K_d,FA_ was performed by measuring the IC_50_ value (inhibition concentration 50%) characterizing each non-fluorescent FA. As DAUDA poorly binds to Cq-FABP (see results section), only cis-parinaric acid (CPA) was used for measuring IC_50_ values. Titration and equilibrium displacement experiments using CPA were performed in buffer C (supplemented with 10% DMSO (v/v)). For the determination of IC_50_ values, the number of complexes corresponding to [CPA] = 7 µM and [Cq-FABP] = 5 µM was varied by increasing the concentration of non-fluorescent FA. The IC_50_ value corresponds to the concentration of non-fluorescent FA that displaces 50% of CPA from the fatty acid binding site of Cq-FABP and is related to K_d,FA_ through the Cheng-Prusoff relationship:
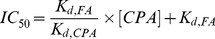
(2)


### Titration experiments using a fluorescently labeled fatty acid: Steady-state fluorescence anisotropy measurements

The binding of 4,4-difluoro-5, 7-dimethyl-4-bora-3a, 4a-diaza-s-indacene-3-hexadecanoic acid (BODIPY-C16) to Cq-FABP was investigated by measuring the steady-state fluorescence anisotropy parameter using a Beacon instrument (PanVera, Madison, USA), in a cell thermostatically held at 25°C. The excitation and emission bandpass filters were centered at 488 nm (±6 nm) and 525 nm (±15 nm), respectively. Titration experiments were performed in buffer C (supplemented with 10% DMSO (v/v)) by maintening a constant concentration of BODIPY-C16 (200 nM) and by adding increasing concentrations of Cq-FABP. Using a vertical direction for the polarized excitation source, the steady-state fluorescence anisotropy (r) was calculated according to:
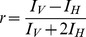
(3)where I_V_ and I_H_ correspond to the parallel (vertical) and perpendicular (horizontal) fluorescence emission intensity components, respectively. The fraction of BODIPY-C16/Cq-FABP complexes (*i.e.* the fraction of BODIPY-C16 bound to Cq-FABP), f_b_, was calculated using the following relationship:
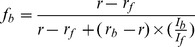
(4)where r is the measured fluorescence anisotropy value, r_f_ and r_b_ correspond to the fluorescence anisotropy values of free BODIPY-C16 (ligand in the free state, measured in the absence of Cq-FABP) and Cq-FABP-bound BODIPY-C16 (ligand in the bound state, corresponding to the maximum anisotropy value found at the plateau), respectively. This relationship takes into account the change in the fluorescence intensity of BODIPY-C16 observed along the titration (typically, the fluorescence intensity of BODIPY-C16 in the bound state, I_b_, was found to be 40% lower than the one characterizing the ligand in the free state, I_f_). The K_d_ characterizing the BODIPY-C16/Cq-FABP complex (K_d,C16_) was calculated by fitting the plot of f_b_ versus [Cq-FABP] using the Graphpad Prism 5.0 software.

The fluorescence anisotropy-based assay was also used in competition experiments to calculate the K_d_ values (K_d,FA_) characterizing non-fluorescent fatty acids. The measurement of the apparent K_d_ value characterizing the BODIPY-C16/Cq-FABP complex (K_d_^app^_,C16_) under different concentration conditions for the non-fluorescent FA allows the calculation of K_d,FA_ according to:
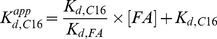
(5)where K_d,C16_ corresponds to the K_d_ characterizing the BODIPY-C16/Cq-FABP complex (in the absence of any non-fluorescent FA) and K_d_^app^_,C16_ corresponds to the apparent dissociation constant obtained for a given concentration of non-fluorescent FA. The calculation of K_d,FA_ was also performed in an independent manner by measuring the IC_50_ value characterizing each non-fluorescent FA. In this approach, the number of complexes corresponding to [C16] = 200 nM and [Cq-FABP] = 5 µM was varied by increasing the concentration of non-fluorescent FA. The IC_50_ value corresponds to the concentration of non-fluorescent FA that displaces 50% of C16 from the fatty acid binding site of Cq-FABP and is related to K_d,FA_ through the Cheng-Prusoff relationship:
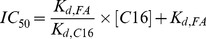
(6)


### Time-resolved fluorescence experiments

The distribution of fluorescence lifetimes of tryptophan was determined by monitoring the intensity decay, I(t), using the single-photon counting technique [27]–[31]. Briefly, the excitation light pulse source was a Ti:sapphire laser (Mai Tai femtosecond laser, Spectra Physics, Mountain View, CA, USA) associated with a third harmonic generator tuned to 296 nm. The laser frequency was reduced from 80 to 8 MHz using a pulse picker (Spectra Physics). Fluorescence emission was detected by a microchannel plate photomultiplier (Hamamatsu model R3809U-05) through a monochromator (ARC SpectraPro-150) set at 340 nm (Δλ = 15 nm) with the emission polarizer set at the magic angle (54.7°). A time-correlated single-photon counting SPC-430 card (Becker–Hickl, Berlin, Germany) was used for the acquisition. Experiments were performed at 25°C using 5 µM Cq-FABP in buffer C. The average fluorescence lifetime values (τ_m_), as reported in Table 1, were calculated from the lifetime distributions recovered by the maximum entropy method [28], [29], [32]. For example, Cq-FABP alone was characterized by three fluorescence lifetimes (τ_1_ = 0.6 ns (6%), τ_2_ = 2.67 ns (53%), τ_3_ = 3.89 (41%); τ_m_ = 3.04 ns).

**Table 1:** Average fluorescence lifetimes of the single Cq-FABP tryptophan under different ANS and PA concentration conditions.

		Average fluorescence Lifetime, τ_m_ (ns)	X^2^	FRET efficiency (%)^a^
1	5 µM Cq-FABP	3.04	1.1330	-
2	5 µM Cq-FABP + 70 µM PA	2.96	1.2624	-
3	5 µM Cq-FABP + 5 µM ANS	2.85	1.2380	6.2
4	5 µM Cq-FABP + 10 µM ANS	2.75	1.3958	9.5
5	5 µM Cq-FABP + 50 µM ANS	2.39	1.1047	21.4
6	5 µM Cq-FABP + 50 µM ANS + 70 µM PA	2.77	1.2535	8.9
7	5 µM Cq-FABP + 200 µM ANS	2.00	1.0655	34.2
8	5 µM Cq-FABP + 200 µM ANS + 70 µM PA	2.34	1.0245	23

a %FRET = 100×[1−(τ_m,D-A_/τ_m_)] where τ_m_ and τ_m,D-A_ correspond to the average fluorescence lifetime values of the donor (Trp) alone and in the presence of the acceptor (ANS), respectively.

## Results

We report the cloning and the characterization of a new member of the FABP family, designated Cq-FABP. The full-length cDNA was cloned from the hepatopancreas of red claw crayfish *Cherax quadricarinatus* and contained an open reading frame (ORF) of 396 base pairs corresponding to a 15-kDa protein (132 amino acids), similar in size to other FABP family members (Fig. 1A). Homologies between Cq-FABP and most of other decapoda FABPs were relatively moderate, *i.e.* comprised between 50% and 60%, with the highest homology obtained with *Pacifastacus leniusculus* (90.9%) (Fig. 1B). Homologies between Cq-FABP and human FABPs were lower (as found when comparing other decapoda and human FABPs – not shown), with the highest homologies found with B- (brain; 40.9%), H- and M-FABPs (heart and muscle, respectively; 38.6%) and the lowest homology found with L-FABP (liver; 31.5%) (Fig. 1B). Transcription profile analysis of Cq-FABP mRNA in different tissues of *C. quadricarinatus*, as performed by qRT-PCR (Fig. S1A in Supplementary Material), indicates a high expression level of Cq-FABP mRNA in hepatopancreas, the main digestive and storage organ for lipids which are further transported via the hemolymph to reproductive organs (*e.g.* ovary in females). A moderate expression level (50–2,000-fold lower than hepatopancreas) was found in intestine, ovary, antennal gland, hemolymph and gills, while a low or very low expression level was found in stomach (10,000-fold lower than hepatopancreas) and testis (100,000-fold lower than hepatopancreas). Moreover, the expression level in hepatopancreas was found to be strongly dependent on the dietary lipid source with the highest and the lowest expression level found using commercial feed (characterized by the highest amount of long chain FA) and pork lard (characterized by a lack of long chain FA), respectively (Fig. S1B in Supplementary Material), suggesting an overall up-regulation mechanism of Cq-FABP gene expression by long chain FAs.

**Figure 2:**
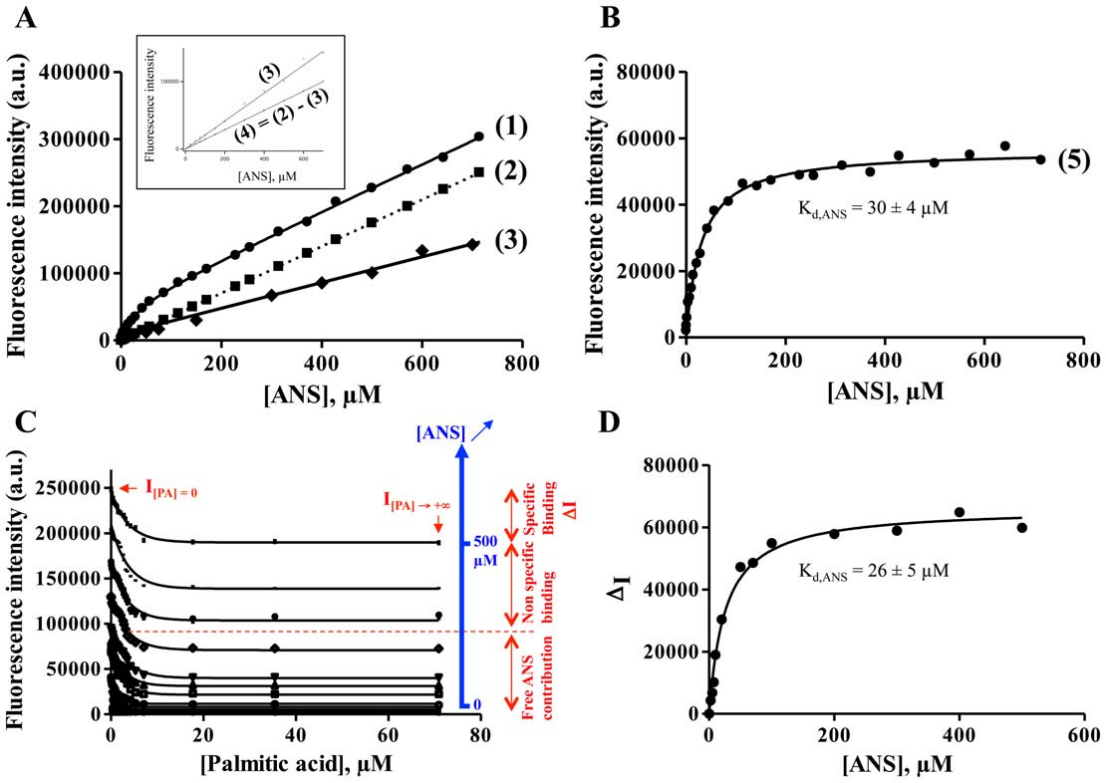
Binding of ANS to Cq-FABP. (**A**) The fluorescence emission intensity of ANS at 25°C (λ_exc_ = 350 nm) was plotted as a function of [ANS] in buffer C containing 5 µM Cq-FABP (curve 1) or in buffer C without protein (curve 3). The curve 1 represents the total intensity (I_total_) - after correction for the inner-filter effect - that accounts for both the “specific” and “non specific” signals (see text in the Results section). The calculated curve 2 represents a straight line having a slope equal to the one calculated in the linear part of curve 1 and that passes through the origin (0,0). This curve accounts for the “non specific” contributions in the ANS emission intensity: (i) the part of the emission fluorescence that originates from free ANS (I_free_) along the titration curve, corresponding to the experimental curve 3 (also reported in the inset), and (ii) the enhancement of the ANS fluorescence intensity due to the “non specific” binding (I_non spec_), corresponding to the calculated curve 4 (inset). (**B**) Resulting binding isotherm (curve 5) after removing the different “non specific” contributions from the overall binding curve: I_spec_ = I_total_ - I_free_ - I_non spec_ or curve 5 = curve 1–curve 2 = curve 1−(curve 3+curve 4). The calculated K_d_ (30 µM) is related to the specific binding of ANS to the fatty acid binding site. (**C**) The fluorescence emission intensity of ANS (I_total_) was plotted against [PA] (after correction for the inner filter effect). Several ANS concentrations were tested (from 0 to 500 µM (blue arrow): 0, 2.5, 5, 7.5, 10, 20, 50, 70, 100, 200, 300, 400, 500 µM) with 5 µM Cq-FABP. The dashed line in red represents the fluorescence intensity level, as determined experimentally, accounting for free ANS under condition of [ANS] = 500 µM. ΔI corresponds to the displacement amplitude (ΔI = I_[PA] = 0_−I_[PA]->+∞_), related to the “specific binding” of ANS (an example corresponding to 500 µM ANS is shown in red color). ΔI was plotted against [ANS] (panel (**D**)).

In order to characterize the binding of several non-fluorescent fatty acids to Cq-FABP, we first determined their apparent affinities indirectly by an equilibrium displacement approach using ANS. ANS is weakly fluorescent when free in solution, however a recovery of the fluorescent signal can be observed upon binding of ANS to hydrophobic cavities of proteins [27]. This approach allows to probe the binding of non-fluorescent fatty acids to FABPs since this binding process is associated with a substantial loss of ANS fluorescence [33], [34]. ANS binding properties of the Cq-FABP were then first investigated. As shown in Fig. 2A, the fluorescence intensity of ANS continuously increased upon binding to Cq-FABP. The experimental curve (curve 1) was clearly biphasic, with a hyperbolic phase followed by a linear phase. The curve 2 is a straight line that displays a slope equal to the linear phase of curve 1 and passes through the origin. The non saturable linear phase appears to be composite and originates in two major contributions. Indeed, the first one is related to the fluorescence emission of free ANS which is not negligible, especially under high concentration conditions (see experimental curve 3 in Fig. 2A; the slope is equivalent to the correction parameter q used in [33]). However, the slope of curve 3 was found to be significantly lower than the slope of curve 2 and the remaining apparent non saturable signal, resulting in curve 4 (Fig. 2A, inset), probably accounts for the “non-specific” binding of ANS to Cq-FABP (in contrast to the “specific” binding that corresponds the ANS binding in the fatty acid binding site) (see below). Thus, the curve 2 accounts for the fluorescence intensities of both free ANS (I_free_) and ANS bound to “non specific” sites (I_non-spec_) and its contribution was removed from the overall binding curve (curve 1), resulting in the hyperbolic curve 5 shown in Fig. 2B that accounts for the binding of ANS to the fatty acid binding site (I_spec_ = I_total_−I_free_−I_non-spec_). The corresponding K_d_ value (K_d,ANS_) was found to be equal to 30 µM.

To confirm that only a part of ANS bound to Cq-FABP actually occupies the fatty acid binding site and, consequently, may inhibit the palmitic acid (PA) binding in a competitive manner, increasing concentrations of PA were added to preformed Cq-FABP/ANS complexes under several ANS concentration conditions (from 0 to 500 µM) and the fluorescence emission intensity of ANS was plotted as a function of [PA] (Fig. 2C). In this experiment, only the correction for the inner filter effect was applied. The starting intensity values (I_[PA] = 0_) increased when ANS concentration increased, according to Fig. 2A. Increasing concentrations of PA displaced ANS but only to a limited extent since I_[PA]->+∞_ did not reach 0; thus, the I_[PA]->+∞_ value represents the part of the fluorescence signal which is not concerned by the competitive displacement. The I_[PA]->+∞_ value was related to contributions of both free ANS (this contribution is explicitly represented by a dashed line in Fig. 2C for the highest ANS concentration: 500 µM) and “non specific” binding of ANS. Importantly, the total amplitude related to the competitive displacement (ΔI = I_[PA] = 0_−I_[PA]->+∞_) continuously increased as a function of [ANS] and was saturable (Fig. 2D), indicating that the maximal amount of ANS removed upon binding to PA represents ANS molecules initially bound to fatty acid binding sites. Note that the estimated K_d,ANS_ value extracted from Fig. 2D (26 µM) was consistent with the value determined above (30 µM).

**Figure 3:**
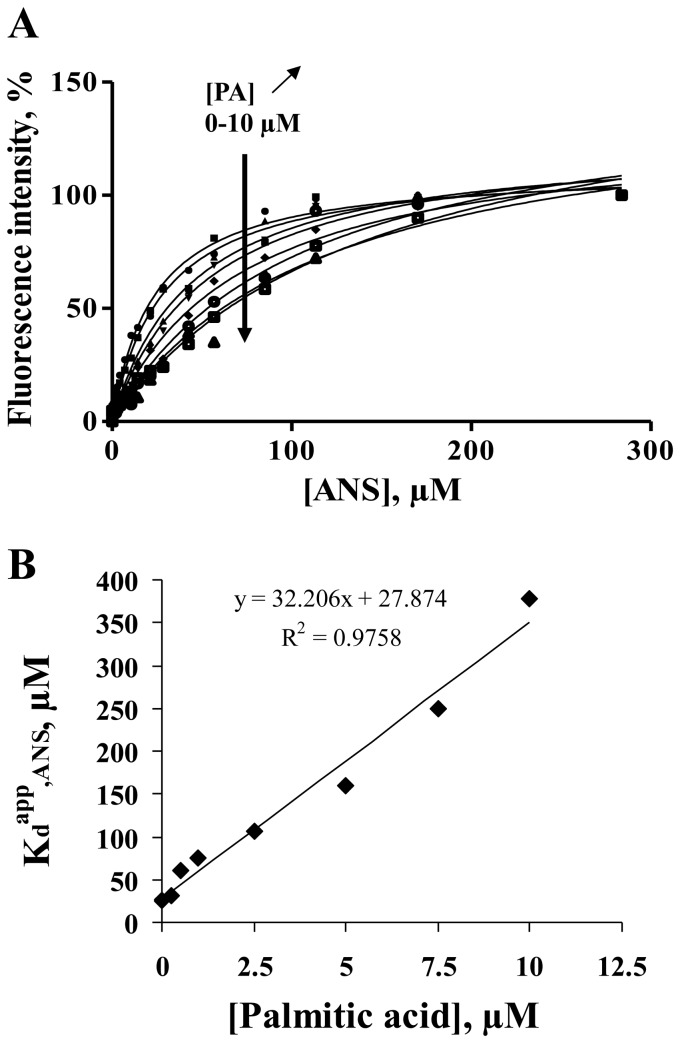
Binding of palmitic acid (PA) to Cq-FABP: competition experiments between PA and ANS. (**A**) Binding of ANS to the fatty acid binding site in the presence of increasing concentrations of PA (after the double correction in order to eliminate the inner filter effect and the “non specific” signal from ANS ( = free ANS + “non specific” binding) as mentioned in Fig. 2). Experimental conditions were similar to those described in the legend of Fig. 2. The concentrations used for PA were: 0, 0.25, 0.5, 1, 2.5, 5, 7.5 and 10 µM. The hyperbolic curves were fitted to determine the apparent K_d_ value of ANS (K_d_^app^_,ANS_). (**B**) K_d_^app^_,ANS_ as a function of PA concentration. The slope represents the K_d,ANS_/K_d,PA_ ratio while the y-intercept represents K_d,ANS_, according to Eq. 1.

The procedure mentioned in Fig. 2A–B was next applied in competition experiments using increasing concentrations of PA, in order to determine the dissociation constant characterizing the Cq-FABP/PA interaction. After corrections for the inner filter effect and “non specific” emission signals of ANS, the binding isotherms - measured for various PA concentrations - showed an increase in the apparent K_d_ value of ANS (K_d_^app^_,ANS_) as a function of PA concentration (up to 10 µM) (Fig. 3A). It is important to note that the linear phases characterizing all the curves, after correction for the inner filter effect but before correction for the “non specific” emission signals of ANS (equivalent to curve 1 in Fig. 2A), displayed similar slopes (data not shown), reinforcing the idea that the linear phase mainly accounts for the fluorescence of free ANS as well as for the “non specific” binding. Indeed, beyond 10 µM PA, no hyperbolic phase was observed when plotting the fluorescence emission intensity as a function of ANS concentration, only a linear dependency was evidenced (data not shown). This indicates that, after saturation of the fatty acid binding site by PA, the further observed increase in the fluorescence emission intensity of ANS was mainly due to the above-mentioned non specific contributions (including free ANS and the “non specific” binding mode of ANS). The plot of K_d_^app^_,ANS_ as a function of PA concentration was found to be linear (Fig. 3B), demonstrating a competitive mode of binding for ANS and PA in the fatty acid binding site. The K_d,PA_ and K_d,ANS_ values were then determined by linear regression. First, the K_d,ANS_ value, as determined by the y-intercept (27.8 µM), was consistent with the values determined above (26–30 µM). Second, the slope value ( = K_d,ANS_/K_d,PA_) led to a K_d,PA_ value of 1.1 µM.

**Figure 4:**
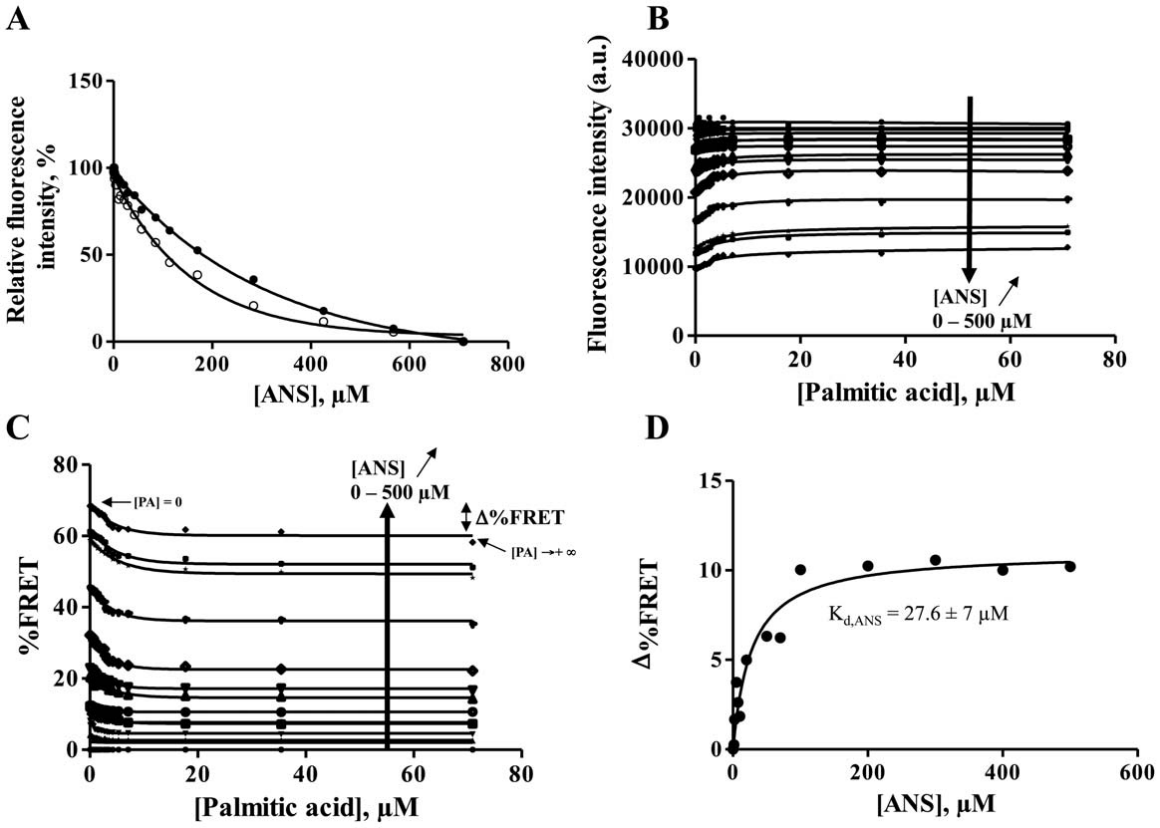
FRET between the W49 residue and ANS for monitoring the ANS binding to Cq-FABP. (**A**) The fluorescence emission of the Trp residue (excitation at 296 nm) was quenched upon addition of increasing concentrations of ANS, in the absence (white circles) or presence (black circles) of 10 µM PA. The fluorescence intensity of Trp (panel (**B**)) or the FRET efficiency (%FRET; panel (**C**)) was plotted as a function of increasing PA concentrations, in the presence of various initial concentrations of ANS: 0, 2.5, 5, 7.5, 10, 20, 50, 70, 100, 200, 300, 400 and 500 µM. %FRET = 100×[1−(I_[ANS]_/I_[ANS] = 0_)] where I_[ANS] = 0_ and I_[ANS]_ correspond to steady-state fluorescence intensity values of the donor alone (W49 residue) and in the presence of the acceptor (ANS), respectively. (**D**) Δ_%FRET_ as a function of ANS concentration. Δ_%FRET_ corresponds to the amplitude of the FRET efficiency between [PA]_0_ and [PA]_+∞_ (as indicated in Fig. 4C for the highest ANS concentration, *i.e.* 500 µM), and is related to the quenching of the W49 residue by ANS bound to the fatty acid binding site only. The deduced K_d,ANS_ value was found to be 27.6 µM.

Next, we used a FRET assay to address the binding of PA to Cq-FABP as (i) a single tryptophan residue (number 49) is present in the protein, close to the fatty acid binding site (based on protein structure homology modeling using SWISS-MODEL [35]; see Fig. S2 in Supplementary Material) and (ii) a strong spectral overlap occurs between the emission of the Trp residue (donor) and the excitation of ANS (acceptor) [27], [36]. The binding of ANS was then assessed by measuring the decrease in the steady state emission intensity of Trp. As shown in Fig. 4A, the fluorescence of the Trp residue was efficiently quenched by ANS. The resulting apparent K_d_ value was about 100 µM, a somewhat higher value compared to the one previously determined (in the 26–30 µM range), suggesting that this value may account for both the “specific” and the “non specific” binding or the “non specific” binding alone. The addition of 10 µM PA shifted the quenching profile, showing that the two subpopulations of ANS, bound to the fatty acid binding site and “non specifically” bound to other sites in the Cq-FABP structure, play a role of acceptor and contribute to the quenching of Trp.

In order to discriminate between the “specific” and the “non specific” binding modes of ANS, we analyzed the recovery of Trp fluorescence by PA after ANS-induced quenching. For a given ANS concentration, the addition of increasing concentrations of PA led to a Trp fluorescence recovery (Fig. 4B). This recovery was not total, and was dependent on the initial ANS concentration, confirming that ANS behaves as an acceptor regardless of its position in the Cq-FABP structure, *i.e.* in the “specific” or “non specific” binding site, with the “non specific” subpopulation preventing the total fluorescence recovery process. In contrast, the recovery amplitude specifically accounts for ANS molecules which are displaced upon binding of PA in the “specific” site. The amplitude of the FRET efficiency between [PA]_0_ and [PA]_+∞_ ( = Δ_% FRET_) obtained for a given ANS concentration (see Fig. 4C), was related to the binding of ANS to the fatty acid binding site only. The Δ_% FRET_ value depends on the fraction of saturation related to the binding of ANS to the “specific” site. The plot of Δ_%FRET_ as a function of [ANS] corresponds to a hyperbolic saturation curve which is characterized by a plateau value (Δ_% FRET,max_) of 10% and an estimated K_d_ value of 27.6 for ANS (Fig. 4D), in accordance with other values (26–30 µM).

The fluorescence average lifetime value (τ_m_) of the single Trp residue of Cq-FABP was found to be 3.04 ns and was not strongly influenced by the addition of 70 µM PA (compare lines 1 & 2, Table 1). The addition of increasing concentrations of ANS, from 0 to 200 µM, continuously decreased the τ_m_ value, from 3.04 to 2.00 ns (lines 1, 3–5 and 7), confirming that FRET occurred between the single Trp residue and ANS. PA (70 µM) was added to samples containing 5 µM Cq-FABP+50 or 200 µM ANS. Based on the results shown in Fig. 2C, 70 µM of PA are sufficient to fully displace ANS from the fatty acid binding site under these two ANS concentration conditions. As expected, the τ_m_ values increased upon addition of PA in the two samples containing a mixture of Cq-FABP and ANS (from 2.39 to 2.77 ns for [ANS] = 50 µM – compare lines 5 & 6 in Table 1 – and from 2.00 to 2.34 ns for [ANS] = 200 µM – compare lines 7 & 8). However, these conditions were not sufficient to observe a total recovery of the initial τ_m_ value, 3.04 ns, as measured in the absence of acceptor. Δ_%FRET,max_ values, based on lifetime values (Δ_%FRET,max_ = 21.4%–8.9% = 12.5% for [ANS] = 50 µM (lines 5 & 6 in Table 1) and Δ_%FRET,max_ = 34.2%−23% = 11.2% for [ANS] = 200 µM (lines 7 & 8)), were comparable to the value based on steady-state fluorescence intensities (10%), as determined in the previous section (Fig. 4D). This result confirms that the recovery of Trp fluorescence upon addition of PA is upper limited due to the non specific binding of ANS to Cq-FABP.

**Figure 5:**
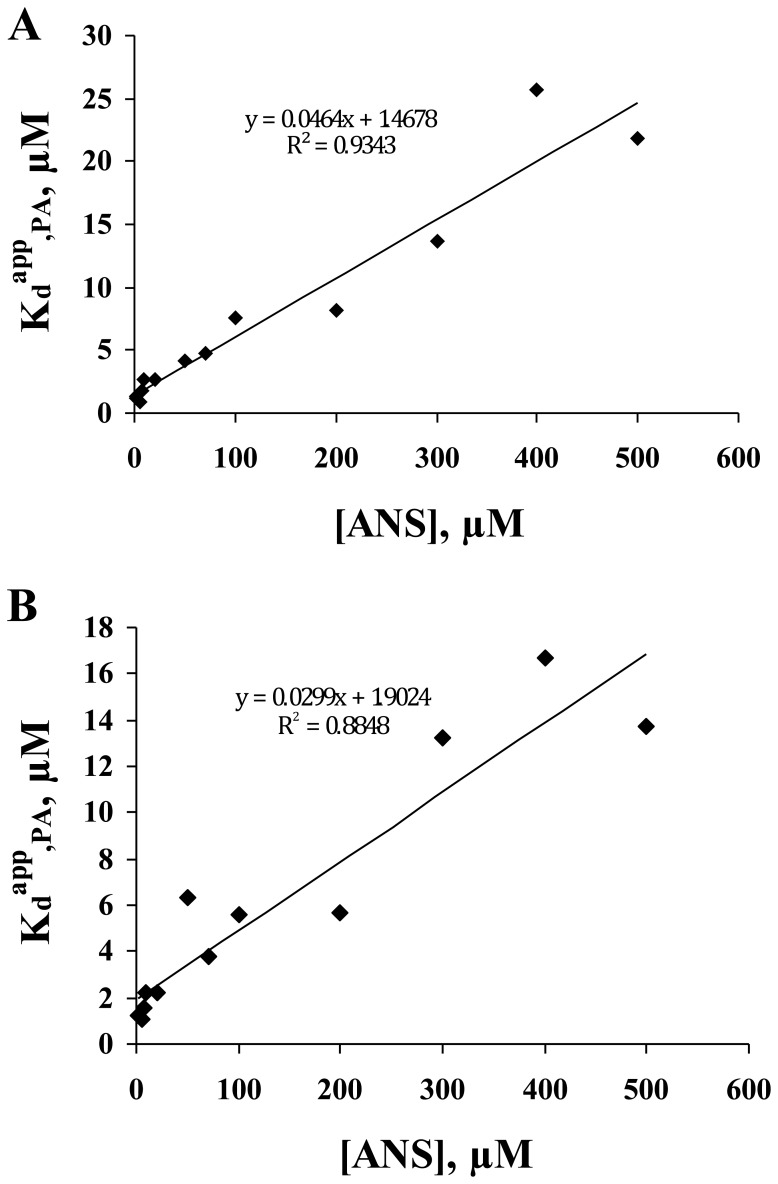
K_d_^app^_,PA_ as a function of ANS concentration. The linear regressions shown in panels (**A**) and (**B**) are related to K_d_^app^_,PA_ values obtained by fitting binding isotherms shown in Fig. 4B and Fig. 4C, respectively. The K_d_^app^_,PA_ value is related to ANS concentration by the following equation: 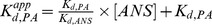.

K_d,PA_ was next calculated using FRET experiments reported in Fig. 4B–C (fluorescence intensity or FRET efficiency, respectively, as a function of PA and ANS concentrations). Using the fluorescence intensity parameter (Fig. 4B), each curve (fluorescence intensity as a function of [PA] for a given ANS concentration) was fitted for the determination of the K_d_^app^,_PA_ value. K_d_^app^,_PA_ was then plotted as a function of [ANS] (Fig. 5A). The y-intercept value (corresponding to K_d,PA_) and the slope value (corresponding to the K_d,PA_/K_d,ANS_ ratio) led to K_d,PA_ values of 1.47 and 1.29 µM, respectively (using an average K_d,ANS_ value of 27.85 µM). A similar approach, but using the FRET efficiency parameter (Fig. 4C), led to K_d,PA_ values of 1.90 and 0.83 µM, respectively (Fig. 5B). All these values were compatible with the one determined by monitoring ANS fluorescence (K_d,PA_ = 1.1 µM in Fig. 3B).

**Figure 6:**
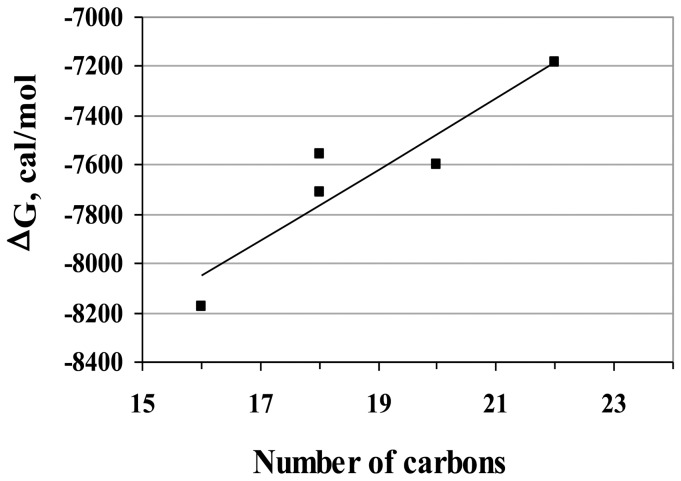
Linear relationship between the fatty acid binding free energy (ΔG) and the number of carbon in the chain. ΔG = −RT×Ln (K_d,FA_). The different K_d,FA_ values are reported in Table 2 and were measured using the procedure described in Fig. 3.

The measurement of the K_d,FA_ parameter is then possible by using one of the two above-mentioned competitive protein binding assay: *(i)* by monitoring the increase of ANS fluorescence signal or *(ii)* by measuring the FRET occurring between Trp and ANS. A special treatment is required in both approaches for accounting for the “non-specific binding” mode of ANS (the fluorescence of free ANS must be corrected in the first approach only). The two approaches yielded comparable K_d,PA_ values, however the R-square of the former (R^2^ = 0.9758 in Fig. 3B) was found to be significantly higher than the R-square of the latter (R^2^ = 0.9343 and 0.8848 in Fig. 5A and 5B, respectively). Therefore, the first procedure was used for a more systematic characterization of various fatty acids (PA, OA, LA, EPA and DHA). The K_d,FA_ values are reported in Table 2 (column 3). These values range from 1.1 µM (for PA) to 5.8 µM (for DHA), accounting for a linear relationship between the fatty acid binding free energy (ΔG) and the length of the carbon chain (Fig. 6).

**Table 2:** K_d_ values characterizing the affinities of different fatty acids for Cq-FABP using fluorescence intensity assays.

Fatty acid	Length of the carbon chain (number of carbon)	K_d,FA_ (µM)^a^	K_d,FA_ (µM)^b^
Palmitic acid (PA)	C16	1.1±0.3	1.8±0.4
Oleic acid (OA)	C18	2.4±0.4	1.3±0.6
linoleic acid (LA)	C18	3.1±0.5	3.3±0.7
Eicosapentaenoic acid (EPA)	C20	2.9±0.3	nd
Docosahexaenoic acid (DHA)	C22	5.8±0.3	4.4±0.8

a calculated from equilibrium displacement experiments using the fluorescence intensity of ANS.

b calculated from the IC_50_ values (see Eq. 2) using the fluorescence intensity of cis-parinaric acid.

nd: not determined.

We wondered whether the above-mentioned problem related to “non specific binding” of ANS also exists when using other fluorescent probes such as DAUDA and cis-parinaric acid [37]–[39]. First, we did not observe significant fluorescence enhancement of DAUDA in the presence of Cq-FABP compared to the intrinsic fluorescence of free DAUDA (Fig. S3A in Supplementary Material), suggesting that DAUDA does not bind or only poorly binds to Cq-FABP. This result was confirmed using the fluorescence anisotropy-based assay with BODIPY-C16 (see below) (inset of Fig. S3A). We found an IC_50_ value of 263 µM (concentration of DAUDA that displaces 50% of BODIPY-C16 from the fatty acid binding site of Cq-FABP), indicating that the Cq-FABP/DAUDA complex is characterized by a very low affinity (apparent K_d_ value of ≈250 µM). In contrast, cis-parinaric acid was characterized by a significant fluorescence enhancement upon binding to Cq-FABP (Fig. S3B in Supplementary Material). The deduced K_d_ value (31.2 µM) was similar to the one found for ANS. Importantly, competitive displacements assays using cis-parinaric acid display a different behavior compared to similar experiments using ANS (Fig. S3C in Supplementary Material). Indeed, increasing concentrations of PA or other non-fluorescent fatty acids fully displaced cis-parinaric acid from Cq-FABP as evidenced by I_[PA]->+∞_ which actually reached 0, indicating that cis-parinaric acid specifically targets the fatty acid binding site of Cq-FABP. This is in sharp contrast to the competition results obtained with ANS from a qualitative point of view, and indicates that only the “specific” binding mode exists when using cis-parinaric acid. The K_d,FA_ values characterizing several fatty acids are reported in Table 2 (column 4). These values are consistent with corresponding values obtained in ANS experiments.

**Figure 7:**
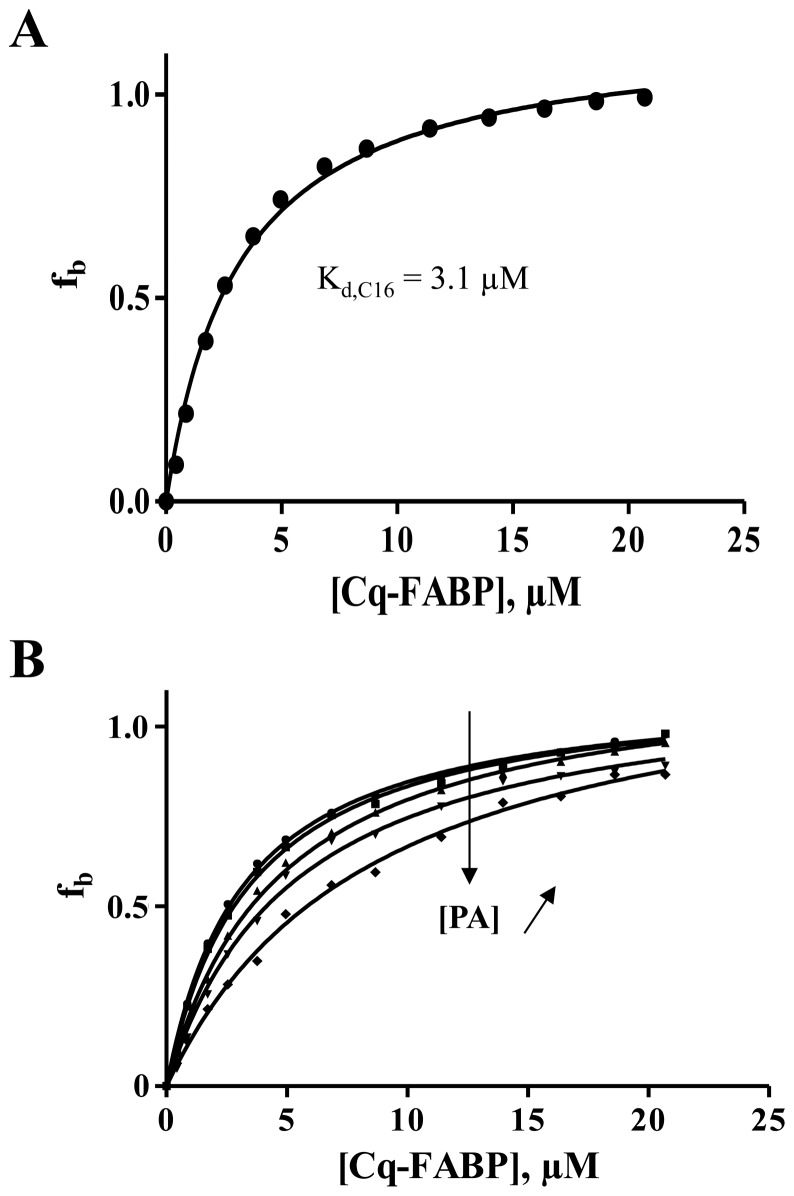
Binding isotherm characterizing the formation of the BODIPY-C16/Cq-FABP complex as measured by steady-state fluorescence anisotropy. (A) 200 nM of BODIPY-C16 was incubated in the presence of increasing concentrations of Cq-FABP. The steady-state fluorescence anisotropy was then measured as described in Materials and Methods. The fraction of ligands in the bound state (f_b_) was calculated according to Eq. 4. (B) Competition experiment showing titration curves obtained at different concentrations of non-fluorescent FA. In the experiment shown, PA was used at 0, 0.5, 1, 2 or 3 µM.

In order to overcome the “non specific binding” problem of ANS-based experiments, we also used a fluorescently labeled fatty acid (BODIPY-C16) [40]. The binding of this fluorescent fatty acid was investigated by monitoring its steady-state fluorescence anisotropy (r) in the presence of varying concentrations of Cq-FABP. Briefly, fluorescence anisotropy measurements are based on the principle of photoselective excitation of a fluorophore by a polarized light, providing information about rotational motions of the fluorescently labeled molecule between photon absorption and emission. Some events such as overall rotational diffusion or flexibility are major causes of light depolarization. The binding of Cq-FABP to BODIPY-C16 increased the r value (from 0.01 to 0.13), by increasing the molecular size of the fluorescent moiety and/or by restricting the internal flexibility of the ligand. This allowed the calculation of the fractional saturation function, f_b_. A typical binding isotherm is shown in Fig. 7A. The corresponding K_d_ value (K_d,C16_) was found to be equal to 3.1 µM.

**Figure 8:**
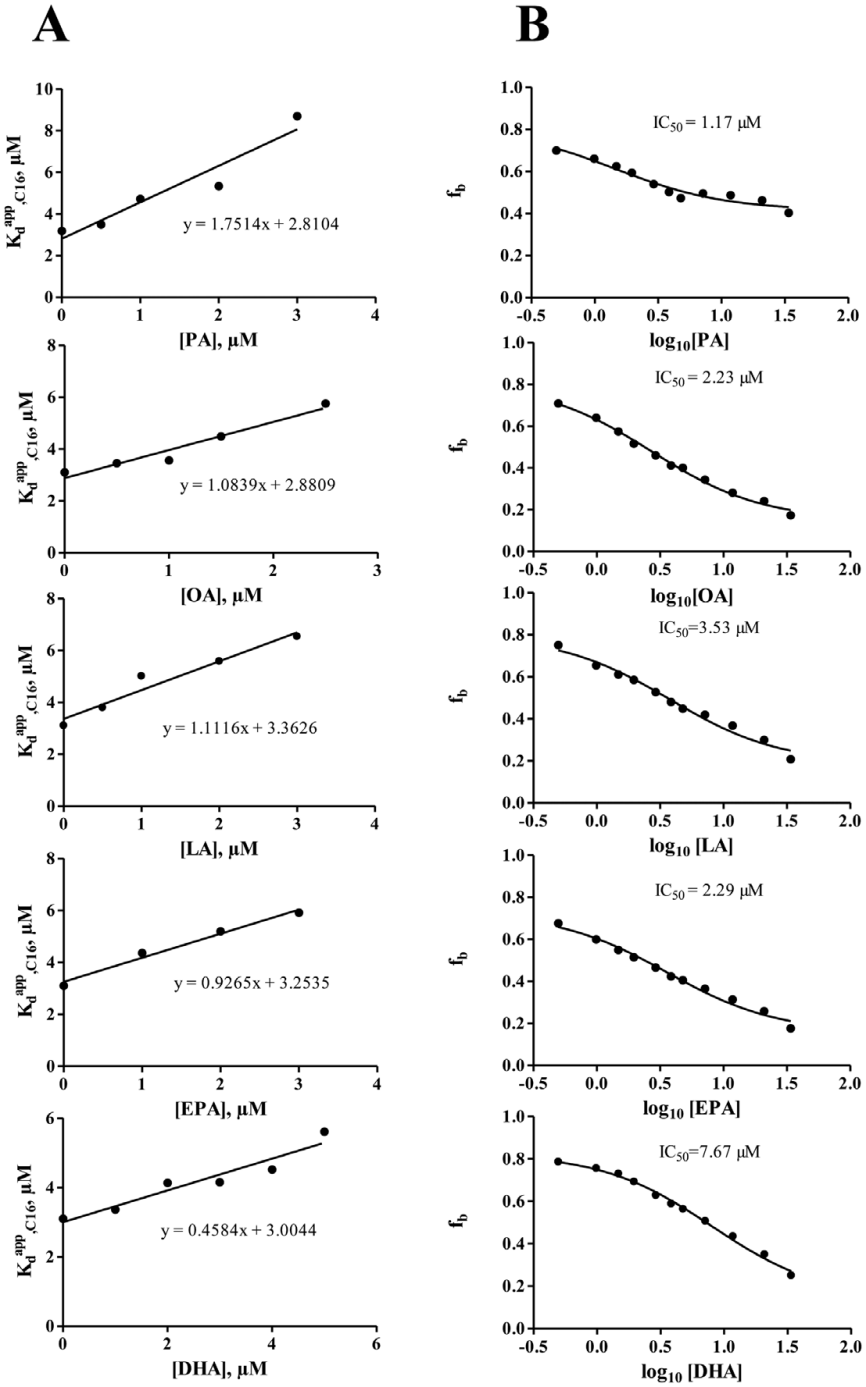
Measurements of K_d,FA_ values using the fluorescence anisotropy assay. (**A**) The fraction of BODIPY-C16 in the bound state (f_b_) as a function of [Cq-FABP] was measured at different concentrations of non-fluorescent FA (a typical experiment is shown in Fig. 7B for PA). The K_d_^app^,_C16_ value (apparent K_d_ value obtained for a given concentration of FA) was then plotted against [FA]. The linear regression was used to calculate K_d,FA_, according to Eq. 5 (the slope corresponds to the K_d,C16_/K_d,FA_ ratio where K_d,C16_ = 3.1 µM, as determined in Fig. 7A). The concentration of BODIPY-C16 was 200 nM. The different K_d,FA_ values are reported in Table 3 (column 2). (**B**) The fraction of BODIPY-C16/Cq-FABP complexes was measured as a function of the concentration of non-fluorescent FA, using 200 nM BODIPY-C16 and 5 µM Cq-FABP. The resulting IC_50_ values were then used for calculations of K_d,FA_ values (Table 3, column 3), according to the Cheng-Prusoff relationship (Eq. 6). The different fatty acids tested were (from top to bottom): palmitic acid (PA), oleic acid (OA), linoleic acid (LA), eicosapentaenoic acid (EPA) and docosahexaenoic acid (DHA).

The affinities of non-fluorescent fatty acids (PA, OA, LA, EPA and DHA) for Cq-FABP were then measured using the fluorescence anisotopy-based assay in two ways. In the first approach, the anisotropy-based titration, as described above, was repeated in the presence of different concentrations of non-fluorescent fatty acids (a typical experiment with PA is shown in Fig. 7B). The corresponding K_d,FA_ values were then calculated using the measured apparent K_d_ value of BODIPY-C16 (K_d_^app^,_C16_), according to Eq. 5 (Fig. 8A and Table 3, column 2). In the second approach, the anisotropy-based assay was conducted in the presence of a given concentration of Cq-FABP (5 µM; corresponding to f_b_≈0.8) and increasing concentrations of non-fluorescent FA (Fig. 8B). The resulting IC_50_ values were used to calculate the K_d,FA_ values according to Eq. 6 (Table 3, column 3). The two approaches led to similar results (compare columns 2 and 3 in Table 3). Moreover, these K_d,FA_ values were found to be in good agreements with those obtained by competition experiments based on the use of ANS or cis-parinaric acid. Using data contained in Table 3, a linear relationship between the fatty acid binding free energy (ΔG) and the length of the carbon chain was observed (data not shown), confirming results shown in Fig. 6. Moreover, most of the tested non-fluorescent fatty acids fully displaced the BODIPY-C16 ligand from the fatty acid binding site of Cq-FABP, leading to a recovery of the low initial level of the r parameter, indicating that, in contrast to the ANS-based assay, the fluorescence anisotropy assay does not suffer from the “non-specific binding” problem, making data analysis easier. Altogether, our data indicate that the fluorescence anisotropy-based assay is reliable and much less time-consuming than the ANS-based assay.

**Table 3:** K_d_ values characterizing the affinities of different fatty acids for Cq-FABP using the fluorescence anisotropy assay.

Fatty acid	K_d,FA_ (µM)^a^	K_d,FA_ (µM)^b^	FA composition (% of total FA) Total^c^	FA composition (% of total FA) Hepatopancreas^d^
Palmitic acid (PA)	1.6±0.3	1.15±0.2	21.05±0.15	24.73±1.61
Oleic acid (OA)	2.6±0.3	2.15±0.4	14.14±0.09	25.21±0.84
linoleic acid (LA)	3.0±0.4	3.4±0.4	14.8±0.17	19.45±1.03
Eicosapentaenoic acid (EPA)	3.5±0.4	2.2±0.4	11.53±0.10	5.23±0.17
Docosahexaenoic acid (DHA)	6.6±0.5	7.4±0.7	10.51±0.11	4.39±0.12

a calculated from the K_d_^app^,_C16_ values, using Eq. 5.

b calculated from the IC_50_ values, using Eq. 6.

c,d from reference [23]. Fatty acid composition (^c^ total; ^d^ in the hepatopancreas) was measured in *C. quadricarinatus*, after a diet of commercial feed as described in [23].

## Discussion

In this work, we present the cloning and a genomic/biochemical characterization of a new invertebrate FABP, named Cq-FABP, from *C. quadricarinatus*, a species of freshwater crayfish. Although its precise physiological function is still unclear, this protein is supposed to play an important role in the intracellular transport of long chain fatty acids and, putatively, is involved in signal transduction in the hepatopancreas of *C. quadricarinatus* [23]. The binding of several fatty acids playing differential effects on the growth and gonad maturation of female *C. quadricarinatus* [23] was then characterized by using four different procedures. Although all these procedures are based on the use of fluorescence and lead to similar results, they are not equivalent in terms of analysis and interpretation. Their advantages and/or limitations are discussed below.

We report on assays for the ligand-binding affinity of Cq-FABP, three of these procedures are based on competitive experiments using the steady-state fluorescence intensity of ANS or cis-parinaric acid while the last one is based on competitive experiments using the steady-state fluorescence anisotropy of the fluorescent fatty acid analog BODIPY-C16. ANS was extensively used in the past for characterizing FABP/ligand interactions because most of the fatty acids commonly used are not fluorescent [33], [34]. ANS displays little or no fluorescence when free in aqueous solvents; however, upon binding to internal hydrophobic cavities of proteins, ANS fluorescence is significantly enhanced. In ANS displacement assays, the release of ANS from FABP, induced by the binding of FA, can be revealed by motoring the decrease in the fluorescence emission intensity of ANS (upon direct excitation of ANS) (“intensity” procedure; Fig. 2 & 3) or by monitoring the recovery of the Trp fluorescence initially quenched by the proximal ANS (“FRET” procedure; Fig. 4 & 5). It is important to note that, in the absence of any structural data for Cq-FABP, it is difficult to certify if the observed quenching of Trp can be simply explained by the energy transfer theory (based on a weak dipole-dipole interaction) or by another mechanism involving exciton coupling as previously suggested for intestinal FABP (I-FABP) [41].

The two above-mentioned procedures obviously need a first correction for the inner-filter effect due to the relatively high ANS and protein concentrations used in the different assays. However, this correction alone is not sufficient in the former procedure. Indeed, as shown in the inset of Fig. 2, the fluorescence of free ANS is not negligible in micromolar concentration ranges. This correction is not always needed depending on the affinities of ANS and fatty acids. In the case of Cq-FABP, the apparent K_d,ANS_ value was 30 µM, significantly higher than the value previously found for other FABPs (in the low micromolar range for I-FABP and L-FABP) [41]–[43], and thus required such a correction. In contrast to the “intensity” procedure, the fluorescence of free ANS is negligible when monitoring the fluorescence emission of Trp in the “FRET” procedure. Nevertheless, both procedures did not exclusively monitor the ANS binding to the fatty acid binding site of Cq-FABP (*i.e.* the “specific” site) and required an additional correction. The “non specific” binding of ANS to secondary binding sites of Cq-FABP is suggested by several independent results: (**i**) The correction for the contribution of free ANS alone was not sufficient to yield a hyperbolic titration curve in the “intensity” procedure (Fig. 2A–B). (**ii**) The displacement of ANS using increasing concentrations of PA was not total (Fig. 2C). Importantly, when the relative change in fluorescence intensity between the sample containing the Cq-FABP/ANS mixture and the corresponding sample containing an excess of PA (ΔI = I_[PA] = 0_−I_[PA]->+∞_) was used for protein titration (Fig. 2D), the measured K_d,ANS_ was similar to the one determined by direct titration (Fig. 2B). (**iii**) A similar behavior was observed using the “FRET” procedure, *i.e.* the enhancement of the steady-state fluorescence of Trp after ANS displacement upon PA binding was not total (Fig. 4). The Δ%FRET parameter was then used for titration and the deduced K_d,ANS_ was consistent with values obtained by other procedures. (**iv**) Accordingly, the presence of an excess of PA was not sufficient to observe a total recovery of the initial fluorescence lifetime of Trp (as measured in absence of ANS) in samples containing different Cq-FABP/ANS mixtures (Table 1), confirming that Trp quenching might be also due to the presence of ANS molecules bound to other sites in Cq-FABP, distinct to the FA binding site.

The amphipathic nature of ANS makes possible both hydrophobic and electrostatic interactions with proteins [44]. The former may account for the ANS binding to protein cavities, including the internal cavity of FABPs, whereas the latter, involving ion pair formation, could explain the non specific binding mode with cationic groups of the protein. Non specific binding of ANS to FABP, due to its amphipathic nature, was rarely observed or reported (the 2∶1 stoichiometry previously reported for the binding of ANS to L-FABP [45], [46] is not considered here as non specific – see below -). However, Prendergast and collaborators have shown that such a non specific binding mode actually occurs with I-FABP that could be counteracted by increasing ionic strength [41]. Here, we show that a non specific binding mode of ANS also exists in Cq-FABP and can be analytically discarded, but only by accomplishing an extensive competition study, *i.e.* by varying concentrations of both ANS and the ligand of interest.

The non specific problem does not occur when monitoring either the steady-state fluorescence intensity of cis-parinaric acid, an intrinsically fluorescent polyunsaturated fatty acid or the steady-state fluorescence anisotropy of BODIPY-C16, a fluorescently labelled fatty acid analog. Due to their chemical nature, these two compounds target specifically the fatty acid binding site, minimizing non specific binding to secondary binding sites. The anisotropy value of BODIPY-C16 significantly increased upon binding to Cq-FABP and, thanks to the additivity law of anisotropy, the titration curve was directly plotted without any correction requirement (filter effect, fluorescence of the free probe, non specific binding in the case of ANS) (Fig. 7). Equilibrium displacement experiments could then be easily conducted (Fig. 8); the measured values of K_d,FA_ were similar to those measured by ANS- or cis-parinaric acid-based assays (Tables 2 and 3).

The comparison of previously reported binding affinities of other FABPs yields to a large range of K_d,FA_ values (from the nanomolar/submicromolar to the low micromolar concentration range), depending on the nature of the FABP and the ligand-binding assay [3], [20], [37]. Here, we found that the affinities of Cq-FABP for natural fatty acids as well as for BODIPY-C16 were in the same range, with K_d_ values in the low micromolar range. In comparison with the two best-characterized human FABPs, I- or L-FABP, which display affinities in the nanomolar range [33], [42], [47], these affinities are significantly lower. ANS also binds Cq-FABP with a moderate affinity (K_d,ANS_ ≈ 30 µM), compared to other values published in the literature (K_d_ = 3–7 µM for I- and L-FABP [41]–[43]; K_d_ = 1.7 µM for FABP4 [6]). Further structural studies are required for understanding these differences. Moreover, we found that the Cq-FABP/DAUDA interaction was characterized by a very low affinity (K_d_ ≈250 µM) as found by others for B-FABP and E-FABP [37], H-FABP [20], [48], M-FABP and A-FABP [20]. It is important to note that this low affinity is not a general feature characterizing all FABPs since L-FABP and I-FABP bind DAUDA with a relative good affinity (K_d_ in the low micromolar range) [20], [37], [48].

Regarding the affinity of Cq-FABP for various FA (*i.e.* PA, OA, LA, EPA, DHA), we found a relationship between the affinity and the length of the carbon chain, with the highest affinity obtained for the shortest FA (PA), suggesting that steric effects primarily influence the interaction of FA in the binding cavity of Cq-FABP. Note that cis-parinaric acid that contains 18 carbon atoms in its aliphatic tail is characterized by a lower affinity than expected based on the above-mentioned relationship, most likely due its unusual conjugated tetraene motif. Our results suggest that, if solubility is generally an important factor for contributing to the overall binding energy - as previously reported for several FABP family members [49], [50] - this is not the main factor to be considered here. Indeed, OA and LA display similar K_d_ values which are significantly higher than the K_d_ of PA (Tables 2 & 3) while PA and OA display similar solubility and these two FAs are five-fold less soluble than LA [49]. A similar relationship was observed with I-FABP but not with L-FABP for which no significant difference was observed between PA, OA, DHA and LA in terms of binding affinity [51]. In the case of I-FABP, it was shown that the differential interaction of several lipophilic compounds is not primarily driven by solubility properties or partition coefficients [43]. Most likely, the influence of the carbon chain length depends on the size of the ligand binding cavity within FABP. This influence could be only evidenced with proteins containing a relatively small cavity. As underlined above for the 2∶1 stoichiometry of the ANS:L-FABP complex, it is well known that most of the ligands – including FAs – bind to L-FABP at two sites [45]. Accordingly, L-FABP cavity is much larger than other FABPs and can accommodate a large size spectrum of ligand [8]; for instance, L- and I-FABP have binding cavities of 610 and 353 Å^2^, respectively [45], [52].

It is generally accepted that FABPs from different tissues display differential affinities for a given FA while FABPs from the same tissue, but from different species, may display similar affinities. Previous studies indicate that the different affinities of FABP types may reflect tissue-specific differences in FA metabolism and function [37], [50]. The tissue distribution of Cq-FABP mRNA was quantified using real-time quantitative RT-PCR. The highest amount was found in the hepatopancreas of *C. quadricarinatus* (Fig. S1A in Supplementary Material). The corresponding gene expression level was strongly dependent on the diet treatment in terms of long chain FA content, with the highest mRNA amount observed with commercial feed (≈ nine-fold higher than that obtained with pork lard as a dietary lipid source) (Fig. S1B in Supplementary Material); commercial feed and pork lard contain the highest and the lowest amount of long chain FA (e.g. C20–22), respectively [23]. This result suggests that the overall amount of long chain FA up-regulates the gene expression level of Cq-FABP, representing a first level of regulation, even though no conclusion about the existence of transcriptional up-regulation by specific FA can be drawn from these data. Such an overall up-regulation of Cq-FABP transcription by long chain FAs could compensate their lower affinities for Cq-FABP compared to FAs with shorter carbon chains (Table 2 & 3).

However, the possibility of a parallel specific down-regulation mechanism cannot be definitively ruled out. Indeed, we have previously shown that linoleic acid (LA) significantly increased the hepatopancreatic vitellogenin (Vg) gene expression [23]. It is considered that optimizing feed formulation enhances egg quality, gonad maturation and fecundity in crustaceans. In particular, vitellogenesis reflects nutrient requirements and is predictable of dietary lipid requirement. Interestingly, in this context, using female red claws in an intermolt stage (to avoid the influence of moulting in Vg synthesis [53]), we found that Vg gene expression was significantly dependent on the diet treatment. Among the different tested lipid sources (fish oil, peanut oil, soybean oil, pork lard, commercial), optimal Vg gene expression level – accounting for ovarian maturation - was observed with soybean oil diet, characterized by high LA content which is the predominant ovarian poly-unsaturated fatty acid (Vg expression levels are reported together with Cq-FABP expression levels in Fig. S1B). Lower Vg gene expression was obtained with fish oil and commercial diets, characterized by high EPA and DHA contents. Anticorrelation of Vg and Cq-FABP expressions is suggested in the present study, at least in the LA context, as the soybean oil diet led to the second weakest expression level of Cq-FABP. Altogether, our results highlight a key role of Cq-FABP in female broodstock quality which strongly influences gonad maturation, fecundity and the quality of both eggs and juveniles, according to previous reports [54]–[56]. We hypothesize a specific LA-mediated down regulation of Cq-FABP at the transcriptional level and that Cq-FABP could negatively regulate the Vg gene expression. Considering that *(i)* FAs can regulate gene expression at the transcriptional level in a FABP-dependent manner [57]–[59], *(ii)* FABPs play a role in gene regulation via Peroxisome proliferator-activated receptors (PPARs), which are nuclear receptors acting as transcription factors of genes involved in lipid and energy metabolism [5], [58], [60], [61], one can reasonably assume that regulation of Vg expression in *C. quadricarinatus* could be mediated by an invertebrate PPAR homologue in a Cq-FABP-dependent manner.

We have previously measured the composition of long chain FA (total or hepatopancreas) after a diet of commercial feed [23]. Interestingly, the levels of the various FA strongly parallel binding affinities (Table 3). Cq-FABP displays lower affinities for long chain unsaturated fatty acids such as DHA (C22) and higher affinities for FAs with shorter carbon chains (C16–18), favoring a preferential intracellular accumulation and transport of the second category of FA. Previous studies have shown that if long chain unsaturated FAs such as EPA and DHA are crucial for marine crustacean species, they are more dispensable in freshwater crustacean species - such as *C. quadricarinatus* - as they can be replaced by other FAs (*e.g.* C18:2 and C18:3) [62], [63]. This is also in accordance with a specific role of Cq-FABP in the regulation of Vg expression, as it was previously shown that EPA and DHA are inefficiently utilized during vitellogenesis [23]. We propose that the binding affinity and selectivity of FABP are adapted to the prevalence of various FAs in the hepatopancreas and could represent therefore a second level of regulation of intracellular trafficking and metabolism of FA in the *C. quadricarinatus* hepatopancreas.

## Supporting Information

Figure S1 **Quantitative RT-PCR analysis of Cq-FABP mRNA.** (**A**) Tissue distribution of Cq-FABP mRNA. qRT-PCR experiments were performed as described in Materials and Methods. The gene expression level in each tissue is expressed as a relative value compared to the lowest expression level (in testis). (**B**) Effect of dietary lipid sources on the Cq-FABP mRNA level in hepatopancreas of female *C. quadricarinatus* (top). The fatty acid composition of different diets is indicated in [23]. The gene expression level of vitellogenin (Vg) was recently studied by one of us under similar conditions [23]. The corresponding values are reported on the bottom histogram. The gene expression levels of Cq-FABP and Vg are expressed as relative values compared to the “fish oil” group (arbitrary reference).
(TIF)

Figure S2 **Cq-FABP structure model obtained by homology modeling.** The proposed three-dimensional structure of Cq-FABP was generated using the SWISS-MODEL Protein Structure and Model Assessment Tools [64]. The possible fatty acid binding site of Cq-FABP (delineated by residues Arg 107, Arg 127 and Tyr 129) is located in the center of the cavity. The single tryptophan residue (Trp 49) used in FRET experiments is explicitly shown. Trp 49 is also located in the cavity, in close proximity to the entrance of the cavity.
(TIF)

Figure S3 **Measurements of K_d,FA_ values using fluorescence intensity-based competitive displacement assay.** (**A**) Emission intensity of DAUDA in the absence (white triangles) or in the presence of 5 µM Cq-FABP (black circles) suggesting that DAUDA does not bind to Cq-FABP. Inset: Competition between DAUDA and BODIPY-C16 for the binding to Cq-FABP using the fluorescence anisotropy assay (see Figures 7 & 8). The fraction of BODIPY-C16/Cq-FABP complexes (f_b_) was measured as a function of DAUDA concentration, using 200 nM BODIPY-C16 and 5 µM Cq-FABP. The resulting IC_50_ value (263 µM) was used for the calculation of the apparent K_d_ value (≈ 250 µM; according to the Cheng-Prusoff relationship) characterizing the Cq-FABP/DAUDA complex. (**B**) Binding isotherm for Cq-FABP/cis parinaric acid interaction (measured in buffer C supplemented with 10% DMSO (v/v)) based on the enhancement of fluorescence emission of cis-parinaric acid upon binding to Cq-FABP (5 µM). (**C**) Competitive displacement assay using Cq-FABP (5 µM), cis-parinaric acid (7 µM) and increasing concentrations of non-fluorescent FA. The resulting IC_50_ values were then used for calculations of K_d,FA_ values (Table 2, column 4), according to the Cheng-Prusoff relationship (Eq. 2). The different fatty acids tested were (from left to right): palmitic acid (PA), oleic acid (OA), linoleic acid (LA), and docosahexaenoic acid (DHA).
(TIF)
