# Supplementary figures and images for: Comparative Study of the Fatty Acid Binding Process of a New FABP from Cherax quadricarinatus by Fluorescence Intensity, Lifetime and Anisotropy
Source: PLoS One. 2012 Dec 21;7(12):e51079. doi: 10.1371/journal.pone.0051079 (PMC3528769; doi:10.1371/journal.pone.0051079)

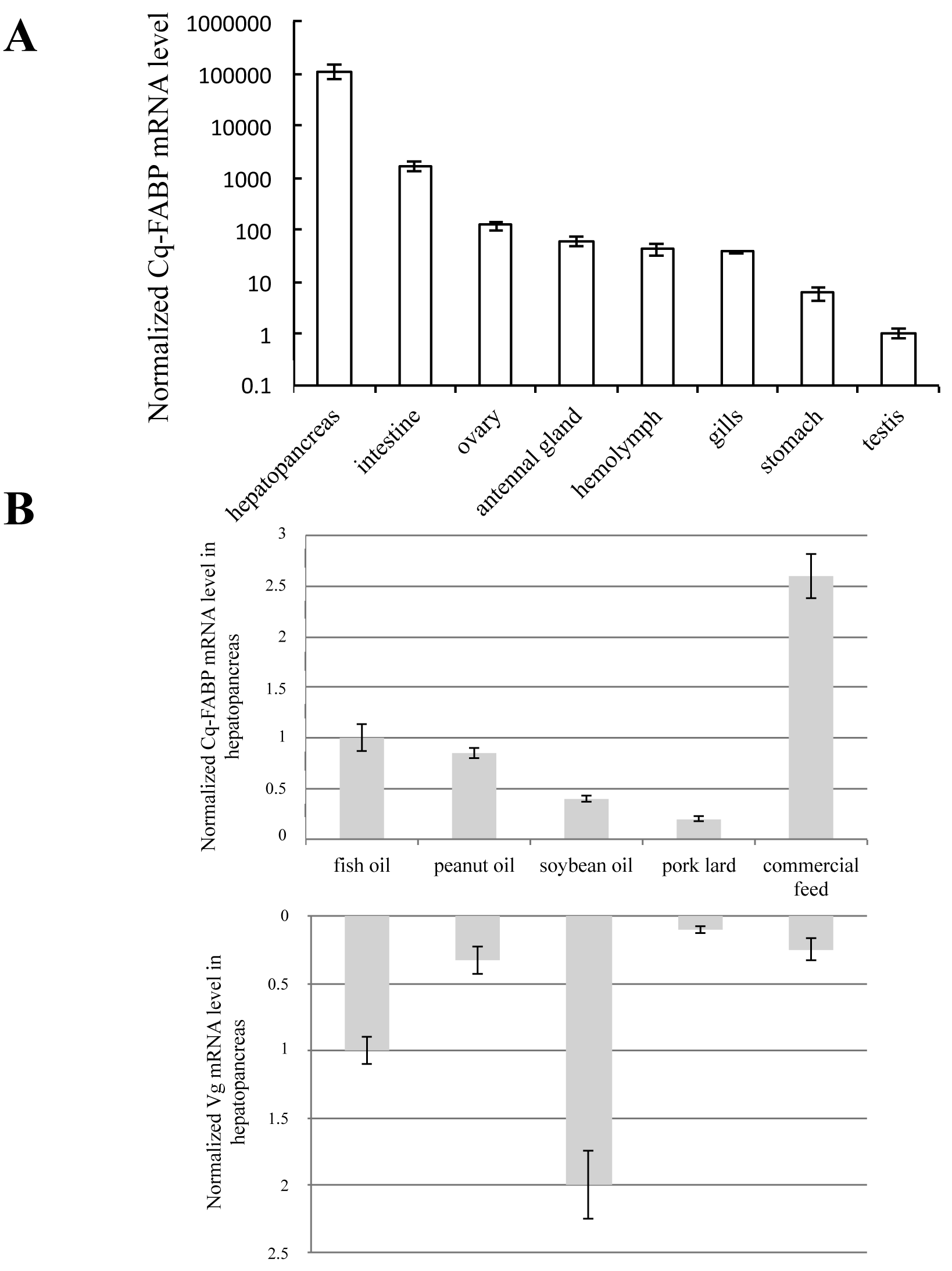

Supplement: Figure S1 — Quantitative RT-PCR analysis of Cq-FABP mRNA. (A) Tissue distribution of Cq-FABP mRNA. qRT-PCR experiments were performed as described in Materials and Methods. The gene expression level in each tissue is expressed as a relative value compared to the lowest expression level (in testis). (B) Effect of dietary lipid sources on the Cq-FABP mRNA level in hepatopancreas of female C. quadricarinatus (top). The fatty acid composition of different diets is indicated in [23]. The gene expression level of vitellogenin (Vg) was recently studied by one of us under similar conditions [23]. The corresponding values are reported on the bottom histogram. The gene expression levels of Cq-FABP and Vg are expressed as relative values compared to the “fish oil” group (arbitrary reference). (TIF) [file pone.0051079.s001.tif]

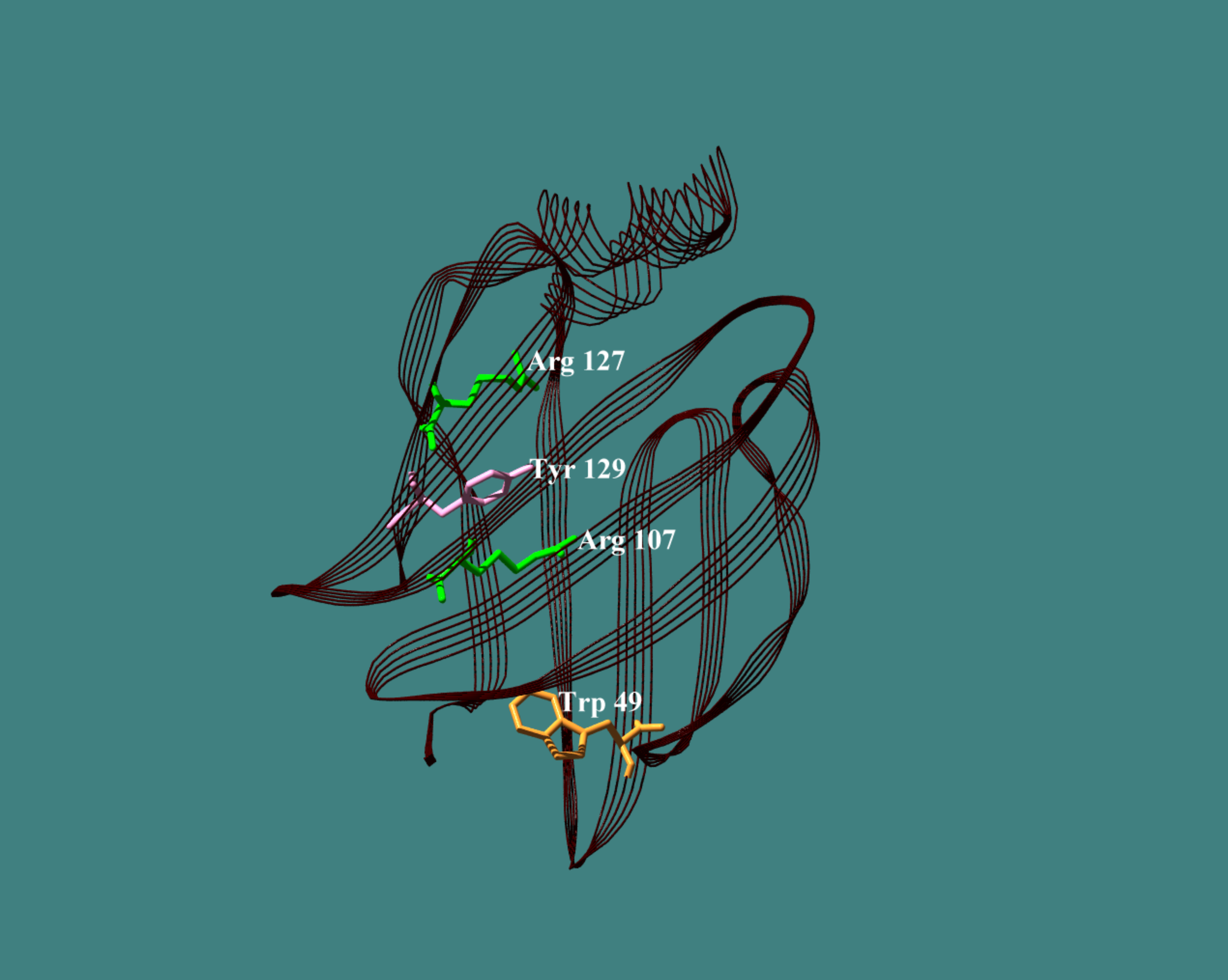

Supplement: Figure S2 — Cq-FABP structure model obtained by homology modeling. The proposed three-dimensional structure of Cq-FABP was generated using the SWISS-MODEL Protein Structure and Model Assessment Tools [64]. The possible fatty acid binding site of Cq-FABP (delineated by residues Arg 107, Arg 127 and Tyr 129) is located in the center of the cavity. The single tryptophan residue (Trp 49) used in FRET experiments is explicitly shown. Trp 49 is also located in the cavity, in close proximity to the entrance of the cavity. (TIF) [file pone.0051079.s002.tif]

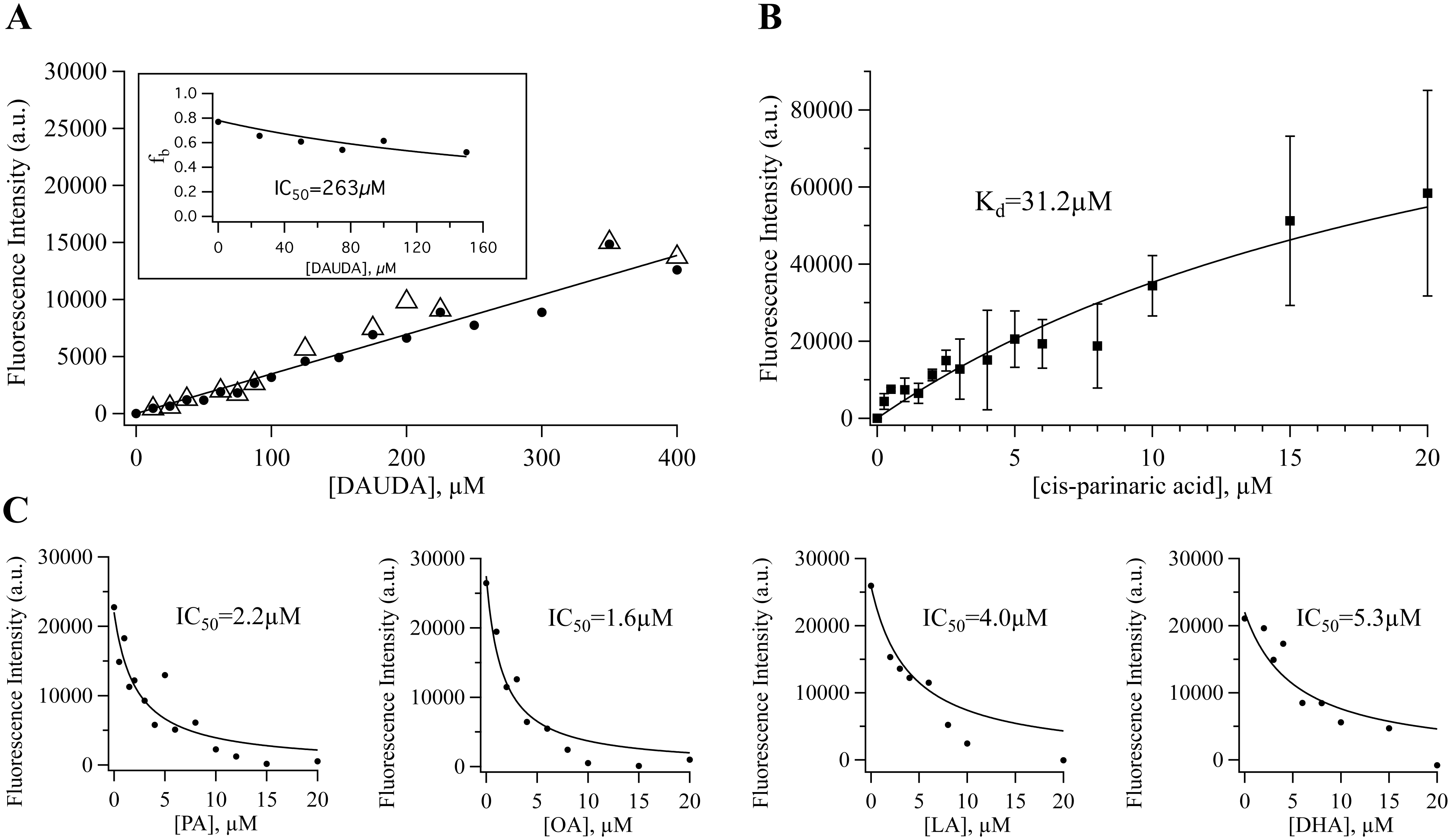

Supplement: Figure S3 — Measurements of Kd,FA values using fluorescence intensity-based competitive displacement assay. (A) Emission intensity of DAUDA in the absence (white triangles) or in the presence of 5 µM Cq-FABP (black circles) suggesting that DAUDA does not bind to Cq-FABP. Inset: Competition between DAUDA and BODIPY-C16 for the binding to Cq-FABP using the fluorescence anisotropy assay (see Figures 7 & 8). The fraction of BODIPY-C16/Cq-FABP complexes (fb) was measured as a function of DAUDA concentration, using 200 nM BODIPY-C16 and 5 µM Cq-FABP. The resulting IC50 value (263 µM) was used for the calculation of the apparent Kd value (≈ 250 µM; according to the Cheng-Prusoff relationship) characterizing the Cq-FABP/DAUDA complex. (B) Binding isotherm for Cq-FABP/cis parinaric acid interaction (measured in buffer C supplemented with 10% DMSO (v/v)) based on the enhancement of fluorescence emission of cis-parinaric acid upon binding to Cq-FABP (5 µM). (C) Competitive displacement assay using Cq-FABP (5 µM), cis-parinaric acid (7 µM) and increasing concentrations of non-fluorescent FA. The resulting IC50 values were then used for calculations of Kd,FA values (Table 2, column 4), according to the Cheng-Prusoff relationship (Eq. 2). The different fatty acids tested were (from left to right): palmitic acid (PA), oleic acid (OA), linoleic acid (LA), and docosahexaenoic acid (DHA). (TIF) [file pone.0051079.s003.tif]
